# Intravenous peramivir vs oral oseltamivir in high‐risk emergency department patients with influenza: Results from a pilot randomized controlled study

**DOI:** 10.1111/irv.12794

**Published:** 2020-10-02

**Authors:** Yu‐Hsiang Hsieh, Andrea F. Dugas, Frank LoVecchio, Breana McBryde, Erin P. Ricketts, Kathryn Saliba‐Shaw, Richard E. Rothman

**Affiliations:** ^1^ Department of Emergency Medicine Johns Hopkins University School of Medicine Baltimore Maryland USA; ^2^ Department of Emergency Medicine University of Arizona College of Medicine Phoenix Arizona USA; ^3^ Division of Infectious Diseases Johns Hopkins University School of Medicine Baltimore Maryland USA

**Keywords:** emergency department, influenza, oseltamivir, peramivir

## Abstract

**Background:**

Peramivir offers a single‐dose intravenous (IV) treatment option for influenza (vs 5‐day oral dosing for oseltamivir). We sought to compare outcomes of emergency department (ED) patients at high risk for influenza complications treated with IV peramivir vs oral oseltamivir.

**Methods:**

During the 2015‐16 and 2016‐17 influenza seasons, adult patients in two US EDs were randomized to either oral oseltamivir or IV peramivir treatment group. Eligibility included positive molecular influenza test; met CDC criteria for antiviral treatment; able to provide informed consent and agree to follow‐up assessment. Outcomes were measured by clinical end‐point indicators, including FLU‐PRO Score, Ordinal Scale, Patient Global Impression on Severity Score, and Karnofsky Performance Scale for 14 days. Non‐inferior *t* test was performed to assess comparative outcomes between the two groups.

**Results:**

Five hundred and seventy‐five (68%) of 847 influenza‐positive patients were approached. Two hundred and eighty‐four met enrollment criteria and 179 were enrolled; of these 95 (53%) were randomized to peramivir, and 84 to oseltamivir. Average FLU‐PRO score at baseline was similar (peramivir: 2.67 vs oseltamivir: 2.52); the score decreased over time for both groups (day 5: peramivir: 1.71 vs oseltamivir: 1.62; day 10: peramivir: 1.48 vs oseltamivir: 1.37; day 14: peramivir: 1.40 vs oseltamivir: 1.33; all *P* < .05 for significantly non‐inferior). Influenza‐related complications were similar between two groups (All: peramivir: 31% vs oseltamivir: 21%, *P* > .05; pneumonia: peramivir: 11% vs oseltamivir: 14%, *P* > .05).

**Conclusions:**

Clinical outcomes of influenza‐infected patients treated with single‐dose IV peramivir were comparable to those treated with oral oseltamivir, suggesting potential utility of peramivir for influenza‐infected patients in the ED.

## 1 INTRODUCTION

Seasonal influenza causes up to 959 000 hospitalizations and 79 400 deaths in the United States annually since 2010.^1^, ^2^, ^3^ As the frontline of the health care system, emergency departments (EDs) see up to three quarter of a million patients during each influenza season.^4^, ^5^ The Centers for Disease Control and Prevention (CDC) recommends that people infected with influenza should receive antiviral treatment, to decrease the occurrence of severe complications and shorten the course of illness, especially for those at high risk of influenza complications. This includes young children, adults 65 years of age and older, pregnant women, and people with certain co‐morbid medical conditions.^6^, ^7^ Currently, there are four Food and Drug Administration (FDA)‐approved antiviral drugs for treatment of influenza, including three influenza virus neuraminidase inhibitor (oseltamivir, zanamivir, and peramivir) and one polymerase acidic protein inhibitor (baloxavir).^8^

Since the 2003‐2004 influenza season, oseltamivir has been the predominant antiviral drug used for ambulatory care patients, including those who come to the US EDs, with a diagnosis of influenza.^9^ However, requirement for a 5 day, twice a day oral dosing regimen, make alternate antiviral drugs more appealing for both clinicians and patients, particularly those seen in acute care setting such as EDs, where filling and adhering with medications is well known to be challenging. Several alternative, single‐dose medication options remain under investigation for ED use, but each has limitations. Zanamivir, which exists as a powder in an inhaled form in the United States, has similar efficacy to oseltamivir, but it is not generally recommended for people with asthma or chronic obstructive pulmonary disease according to CDC^8^; baloxavir which was recently approved in the United States by FDA in October 24, 2018,^10^ 15 months after the end of our study, is restricted to use in those >12 years old and has not been studied in those >65 years old, pregnant, or lactating women.^8^ In addition, there are concerns of rapid emergence of resistance to the use of baloxavir.^11^, ^12^ These leaves peramivir, which can be used in patients ≥2 years old, as a potential favorable alternative candidate antiviral drug for treating ED patients with influenza.

Peramivir, a neuraminidase inhibitor agent with the same mechanism of action as oseltamivir, has been demonstrated to have activity against both influenza A and B viruses, and shorten duration of influenza symptoms for outpatient adults with uncomplicated influenza.^13^, ^14^ Several studies previously demonstrated both safety and non‐inferiority of peramivir hospitalized patients and outpatients with influenza.^13^, ^15^, ^16^ A multinational, multicenter, double‐blind randomized trial in East Asia showed that the duration of influenza symptoms in adult influenza‐infected patients treated with a single dose of 300 mg of IV peramivir, or 600 mg of peramivir was non‐inferior to that treated with 5‐day twice a day oseltamivir. The incidence of severe adverse events by peramivir was similar to oseltamivir.^13^ de Jong and colleagues conducted a trial in hospitalized patients with suspected influenza randomized to 5‐day treatment with intravenous peramivir (600 mg once daily) or placebo; all received the institution's standard of care treatment. That also showed that no difference in median time to clinical resolution between the two groups. However, there was a trend toward a shorter time to clinical resolution in ≥4 of 5 vital sign abnormalities (temperature, oxygen saturation, respiration rate, heart rate, and systolic blood pressure) for 24 hours, among those who required intensive care who received peramivir (vs oseltamivir).^16^ In another small randomized trial of 92 adult inpatients and outpatients with high‐risk factors, the results also showed that patients treated with single‐dose 600 mg peramivir had similar outcomes with regard to time to reduce fever, total symptom scores, and virus titer as compared to those treated with orally administrated oseltamivir (75 mg, twice per day for 5 days).^15^ Accordingly, CDC includes peramivir as a recommended agent, which can be administered intravenously which should be considered for patients who cannot tolerate or absorb orally or enterically administered oseltamivir.^7^ Given that only one‐dose via intravenous (IV) is required for use of peramivir for influenza treatment, the agent might be a welcome alternate antiviral in acute episodic setting such as EDs; further during future influenza seasons (or during a pandemic) it is possible that selectively increased resistance to oseltamivir (vs peramivir) could occur.^17^ To date, there are no studies comparing the outcome of ED patients treated with peramivir vs oseltamivir patients considered at high risk for influenza complications.

We sought to determine the outcomes and safety of peramivir vs oseltamivir in patients diagnosed in the ED with influenza, who are at high risk for influenza complications according to CDC risk criteria. Data for this analysis were collected from a pilot randomized controlled trial intended to evaluate the practical feasibility of enrolling subjects for influenza therapeutic trials in the ED setting. The outcomes of antiviral treatment were measured using several clinical end‐point indicators, including FLU‐PRO Score, Ordinal Scale, Patient Global Impression on Severity (PGIS) Score, and Karnofsky Performance Scale, collected via patient's daily diaries and phone follow‐ups.

## 2 METHODS

An open‐label randomized controlled clinical trial was conducted at two academic EDs: The Johns Hopkins Hospital (JHH), Baltimore, Maryland, and Maricopa Medical Center (MMC), Phoenix, Arizona. ED patients who tested positive for influenza (see below) during their ED encounter were randomized to receive either oral oseltamivir or IV peramivir antiviral treatment.

Eligible patients were those (a) 18 years or older; (b) with an ED positive influenza test by rapid molecular influenza assay (GeneXpert Flu/RSV; Cepheid); (c) meeting the 2011 CDC criteria for antiviral treatment; (d) with symptoms onset of less than 96 hours; (e) able to provide informed consent; and (f) expressed willingness to comply with all study procedures including follow‐up requirements (completing daily diary logs and available for phone calls with a study coordinator). A patient was considered ineligible if (a) they did not speak or understanding English (JHH); or English or Spanish (MMC site); (b) unable or unwilling to provide informed consent; (c) previously enrolled in the study during the current influenza season; (d) unable to take oral medication; (e) using any neuraminidase inhibitors within the past 7 days; (f) known allergic reaction to neuraminidase inhibitors; (g) pregnant or breastfeeding; and (h) having end‐stage renal disease, end‐stage liver disease, glucose‐6‐phosphate dehydrogenase (G6PD) deficiency, or immunodeficiency. Dedicated trained study coordinators recruited eligible patients 24 hours a day, 7 days a week from 11/2015‐04/2016 (JHH only) to 11/2016‐04/2017 (JHH & MMC). Study coordinators first screened all ED patient charts to identify patients who had a positive laboratory‐confirmed rapid PCR influenza test, then approached potentially eligible patient to gauge their interest in participating in the study and to determine if the patient met eligibility criteria before conducting written informed consent. Potentially eligible patients were approached for the study when the positive result of rapid molecular influenza test came back. All Emergency Medicine physicians were trained by the site PI, on study protocol and procedures, completing and signing a Statement of Investigator, Form FDA 1572. A study trained physician provided written informed consent to patients who were eligible and expressed interested in participating, explaining the risks and benefits of the study to the patient and ensuring that the patient understood all aspects of the study (study coordinators were present with the physician, to assist where needed). Consented patients were randomized to oral oseltamivir or IV peramivir treatment group using an internet‐based computerized randomization system (www.random.org) without a block randomization design but with an intent of 1:1 ratio. The random number generated for each consented patient was an independent event and independent by site. The randomization was not stratified by the study site. Since this study was intended to evaluate the practical feasibility of enrolling subjects for influenza therapeutic trials in the ED setting, the sample size of 50‐150 subjects sample size was determined in collaboration with the funder (Department of Health and Human Services Office of the Assistant Secretary for Preparedness and Response/Biomedical Advanced Research and Development Authority) to be adequate for this pilot effort which is being conducted specifically to examine the feasibility of achieving higher recruitment rates than has historically been achieved in other clinical venues, and the ability to reliably collect useful therapeutic end‐point data from an ED enrollment site.

Both oseltamivir and peramivir were dosed based on creatinine clearance (CrCl) results which was calculated using the Cockcroft Gault equation; 30 mg once daily, 30 mg twice daily or 75 mg twice daily of oseltamivir for 5 days or 100 mg, 200 mg, 600 mg of one‐time IV peramivir. Both groups received the first dose of antiviral treatment in the ED following randomization. For the oseltamivir group, patients were instructed to take the remaining doses on the subsequent 4 days, either inpatient or outpatient, based on disposition from the ED attending. For the peramivir group: for patients who were discharged from the ED, no further study drug was administered; for patients admitted to the hospital from the ED, the inpatient treating provider was given the option to choose to continue administering IV peramivir, based on their clinical discretion. An investigator from the study team gave the inpatient treating provider information about the study, including information on how to continue IV peramivir at the same dose for each subsequent day for up to 4 days. If a participant remained in the hospital beyond 5 days of treatment, and the patient was symptomatically better, treatment stopped. If the patient remained hospitalized after 5 days of treatment and had not improved, the treating provider was given the option to continue IV peramivir daily for another 5‐day course (with consultation as requested from a 24/7 on‐call infectious disease specialist and pharmacist, to assist with decision‐making). Treatment with peramivir was discontinued upon discharge from the hospital for all participants in the IV peramivir arm.

As a secondary objective, we created a repository of residual nasopharyngeal samples from ED patients with suspected influenza illness for purposes of future laboratory analysis of new assays with potential interest for characterizing patients with influenza. Specimens were collected by clinical staff at day 1 (baseline) according to standard of care practice and at day 3 (under a research protocol) using a flocked swab and universal viral transport media. Day 1 specimen was first testing for clinical purposes by Xpert Flu, and the remaining specimen was transported to, frozen and stored at the central study laboratory at JHH for future analysis. For the day 3 specimen, the entire specimen was transported to, frozen and stored at the central laboratory at JHH for future analysis (see below). The study was approved by the IRB at each of the participating institutions. This study was registered as protocol: NCT02609399 at clinicaltrials.gov.

Outcomes of antiviral treatment were measured by the validated FLU‐PRO score,^18^ a 32‐question clinical end‐point indicator (scale 1‐5 for each question) from enrollment (day 1) for 14 days via patients' daily diary. Influenza disease severity was also assessed by PGIS, whereby participants rated their influenza symptoms ranging from no symptom (score 0), mild symptoms (1), moderate symptoms (2), or severe symptoms (3) at the time of enrollment, day 7 and 28.^19^, ^20^ Clinical status of the participants was evaluated by a validated 6‐step Ordinal Scale (1‐6) from the day of ED or hospital discharge as: return to normal activities, 1 point; discharged but not back to normal activities, 2 points; Non‐ICU hospitalization, 3 points; ICU without mechanical ventilation, 4 points; ICU with mechanical ventilation/ ECMO, 5 points; and death, 6 points.^21^ For any patient who was discharged where the status of back to normal activities was unknown for any particular day, the Ordinal Scale for that day was conservatively coded as “2 points”. If the patient reported returning to normal activities the previous day, and reported normal activities the day after then the Ordinal Scale was coded as “1 point”. Physical function of the participants was assessed for 14 days by daily diary reports using the Karnofsky Performance Scale, which ranges from 0 to 100, with higher scores indicating better performance status.^22^ For this pilot feasibility phase of the study, all of follow‐ups after the participant's discharge from the ED or hospital were conducted by study coordinators by phone.

We evaluated specimens from our biorepository for any patients in whom we were able to collect paired nasopharyngeal swab specimens (at both day 1 and 3) using a feature of the Cepheid GeneXpert® Xpress Flu/RSV real‐time PCR assay which permitted us to assess the cycle threshold (C*_t_*) values, as a semi‐quantitative approach to infer influenza viral load from any particular sample. Analysis of C*_t_* values using this approach was demonstrated previously to inversely reflect the amount of influenza viral RNA present in the sample.^23^ A C*_t_* value of 40 for influenza A or B viruses was considered as an undetectable viral load for influenza A or B virus, respectively.

Descriptive data analysis was performed first, followed by chi‐square tests to determine the differences between the two treatment groups with regard to socio‐demographic and clinical characteristics at baseline. Adherence to the assigned treatment regimen was defined as required peramivir or oseltamivir dosages that a patient received recorded in the chart (peramivir or oseltamivir group), or reported during the follow‐up by the patient if he or she was discharged (oseltamivir group). Chi‐square tests were then performed to determine the differences in adherence, complications, and relevant side effects between the two groups. *T* test for non‐inferiority was performed to determine daily outcome measures using the original full dataset. For this, *P* < .05 indicated that the outcome of peramivir treatment was not inferior to that of oseltamivir. To examine the impact of missingness of each outcome of antiviral treatment during the follow‐up, sensitivity analyses were performed. We performed the non‐inferiority tests for 15 multiple‐imputed datasets for each outcome of antiviral treatment by each time point, using the same approach described above. All data analyses were based on intent‐to‐treat analysis.

## 3 RESULTS

**FIGURE 1:**
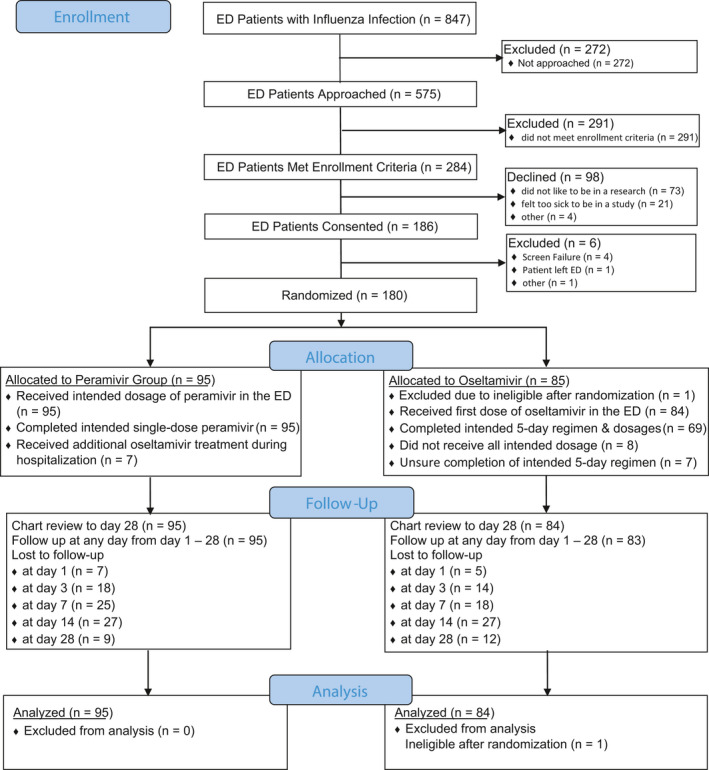
Diagram of study design and patient enrollment

Overall, 847 patients with laboratory‐confirmed influenza were seen at the ED sites during the study period. Among them, 575 (68%) were approached by the study coordinator, 284 (49%) of those met study enrollment criteria. Among those eligible, 186 (65%) provided consent, and 180 were enrolled and randomized (Figure 1). After excluding one patient who was determined to be ineligible following enrollment, a total of 179 ED patients with influenza were analyzed, including 58 and 121 ED patients during influenza season 2015‐2016 and 2016‐2017, respectively. The majority were female (59%) and African American (67%) with a median and mean age of 50 years (interquartile range: 36‐57 years) and 47.4 years (standard deviation: 15.0 years; range 19‐80 years). The most common CDC‐defined higher risk for influenza complications was chronic pulmonary disease (60.3%), followed by intent to admission (38.5%), chronic metabolic disease (31.8%), chronic cardiovascular disease (22.9%), aged of 65 years or older (14.0%), and morbid obesity (14.0%). There were 121 (67.6%) participants with more than one CDC‐defined higher risk. There were more than 50% of enrollees who had onset of symptoms for more than 48 hours (n = 100, 55.9%). 135 (75%) patients were infected with influenza A virus and 44 (25%) with influenza B virus. 95 (53%) patients were randomized to the peramivir treatment arm and 84 to the oseltamivir treatment arm. There were no statistical differences between the peramivir and oseltamivir treatment groups, including co‐morbidities listed by CDC (Table 1). The percentage of the influenza A virus infection was 73.7% in the peramivir group and 77.4% in the oseltamivir group (*P* = .567). The percentage of the inpatient admission in two groups was similar (peramivir: 35.8% vs 36.9%, *P* = .877). All 95 patients in the IV peramivir group received intended dosage of antiviral medication (Figure 1). On the other hand, approximately, 20% of patients who received oseltamivir did not receive the intended antiviral dosage (n = 8, 9.5%) or we did not know their adherence information (n = 7, 8.3%). There was a statistical different between two groups (*P* < .001). There were six patients in the peramivir treatment group who received peramivir in the ED and then received additional oseltamivir treatment during hospitalization. There were seven patients in the peramivir treatment group who received additional peramivir dosages during hospitalization (three with one additional dosage of peramivir, two with two, and two with four) while there was one patient in the oseltamivir treatment group who received one additional dosage of oseltamivir during hospitalization (Figure 1).

**TABLE 1:** Characteristics of 179 emergency department patients with influenza enrolled in the influenza therapeutic study

Characteristics	Category	Total No.	Peramivir group	Oseltamivir group	*P*‐value
		N = 179	N = 95	N = 84	
Age (years)	18‐34	43 (24.0)	22 (23.2)	21 (25.0)	.824
	35‐49	45 (25.1)	25 (26.3)	20 (23.8)	
	50‐64	66 (36.9)	33 (34.7)	33 (39.3)	
	≥65	25 (14.0)	15 (15.8)	10 (11.9)	
Sex	Male	73 (40.8)	37 (38.9)	36 (42.9)	.595
	Female	106 (59.2)	58 (61.1)	48 (57.1)	
Race	African American	120 (67.0)	60 (63.2)	60 (71.4)	.455^a^
	White	50 (27.9)	29 (30.5)	21 (25.0)	
	Other	9 (5.0)	6 (6.3)	3 (3.6)	
Ethnicity	Hispanic	29 (16.2)	19 (20.0)	10 (11.9)	.142
CDC‐defined high risk	Intent to admit to observation unit or admission	69 (38.5)	35 (36.8)	34 (40.5)	.618
	Complications ‐ pneumonia	12 (6.7)	3 (3.2)	9 (10.7)	.069 ^a^
	Age 65 y or greater	25 (14.0)	15 (15.8)	10 (11.9)	.454
	Chronic pulmonary disease	108 (60.3)	59 (62.1)	49 (58.3)	.607
	Chronic cardiovascular disease	41 (22.9)	24 (25.3)	17 (20.2)	.425
	Chronic renal disease	8 (4.5)	4 (4.2)	4 (4.8)	1.000 ^a^
	Chronic hepatic disease	23 (12.8)	12 (12.6)	11 (13.1)	.926
	Chronic hematologic disease	7 (3.9)	3 (3.2)	4 (4.8)	.708 ^a^
	Chronic metabolic disease	57 (31.8)	33 (34.7)	24 (28.6)	.377
	Chronic neurologic disease	22 (12.3)	14 (14.7)	8 (9.5)	.289
	Immunosuppression	15 (8.4)	7 (7.4)	8 (9.5)	.604
	Pregnancy	0 (0.0)	0 (0.0)	0 (0.0)	NC
	Morbid obesity	25 (14.0)	13 (13.7)	12 (14.3)	.908
	Resides in nursing home	2 (1.1)	1 (1.1)	1 (1.2)	1.000 ^a^
	Native American	2 (1.1)	1 (1.1)	1 (1.2)	1.000 ^a^
Influenza vaccination	No vaccination	101 (56.4)	57 (60.0)	44 (52.4)	.415 ^a^
	Within last 2 wk	6 (3.4)	4 (4.2)	2 (2.4)	
	More than 2 wk	72 (40.2)	34 (35.8)	38 (45.2)	
Symptoms	Subjective fever	140 (78.2)	75 (78.9)	65 (77.4)	.800
	Documented fever	71 (39.7)	33 (34.7)	38 (45.2)	.152
	Cough	168 (93.9)	90 (94.7)	78 (92.9)	.601
	Headache	113 (63.1)	62 (65.3)	51 (60.7)	.529
	Short of breath	134 (74.9)	69 (72.6)	65 (77.4)	.465
	Sore throat	89 (49.7)	50 (52.6)	39 (46.4)	.408
	Rhinorrhea	102 (57.0)	57 (60.0)	45 (53.6)	.386
	Congestion	98 (54.7)	49 (51.6)	49 (58.3)	.365
	Sinusitis	72 (40.2)	40 (42.1)	32 (38.1)	.585
Onset of symptoms	Within 2 d	79 (44.1)	39 (41.1)	40 (47.6)	.598
	3 d	46 (25.7)	27 (28.4)	19 (22.6)	
	4 d	54 (30.2)	29 (30.5)	25 (29.8)	
NEWS score	0	18 (10.1)	9 (9.5)	9 (10.7)	.165 ^a^
	1‐3	111 (62.0)	54 (56.8)	57 (67.9)	
	4‐6	41 (22.9)	28 (29.5)	13 (15.5)	
	>6	9 (5.0)	4 (4.2)	5 (6.0)	
ED disposition	Admit	65 (36.3)	34 (35.8)	31 (36.9)	.877

Abbreviation: NC: Not calculated.

^a^ Fisher's exact test.

**FIGURE 2:**
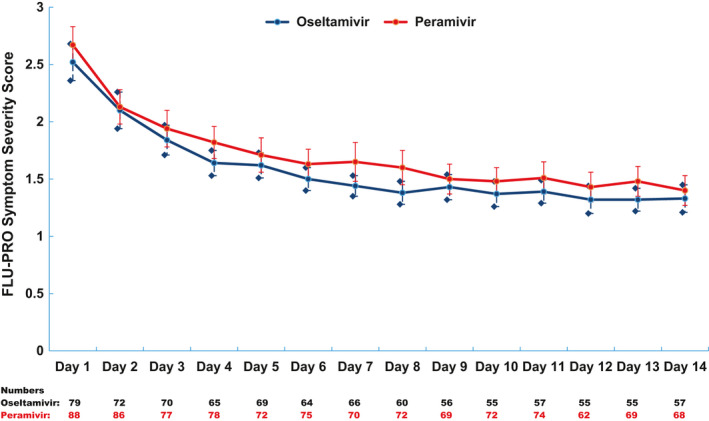
FLU‐PRO symptom severity score for the 14 d of follow‐up by antiviral treatment group. The blue diamonds represent the lower and upper bound of a 95% confidence interval of each point estimate of the FLU‐PRO value of patients in the oseltamivir group; the red bars represent the lower and upper bound of a 95% confidence interval of each point estimate of the FLU‐PRO value of patients in the peramivir group

**FIGURE 3:**
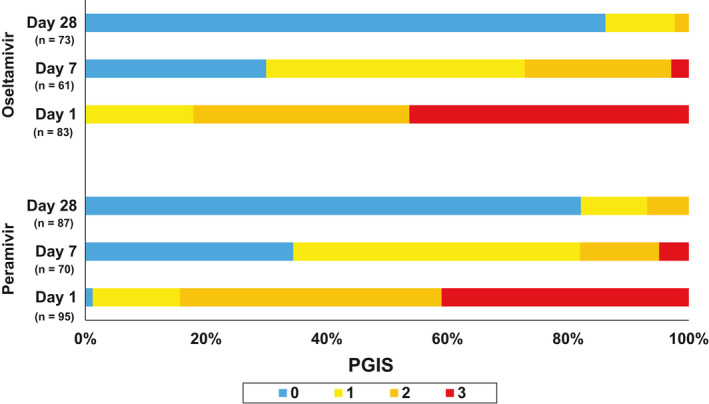
Patient global impression of severity

**FIGURE 4:**
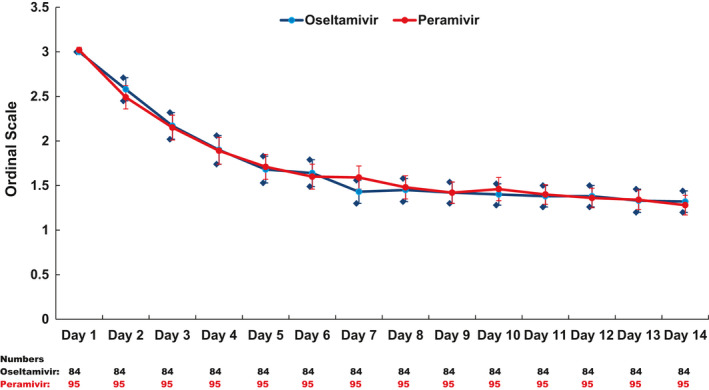
Ordinal scale for the 14 d of follow‐up by antiviral treatment group. The blue diamonds represent the lower and upper bound of a 95% confidence interval of each point estimate of the Ordianl Scale of patients in the oseltamivir group; the red bars represent the lower and upper bound of a 95% confidence interval of each point estimate of the Ordianl Scale value of patients in the peramivir group

**FIGURE 5:**
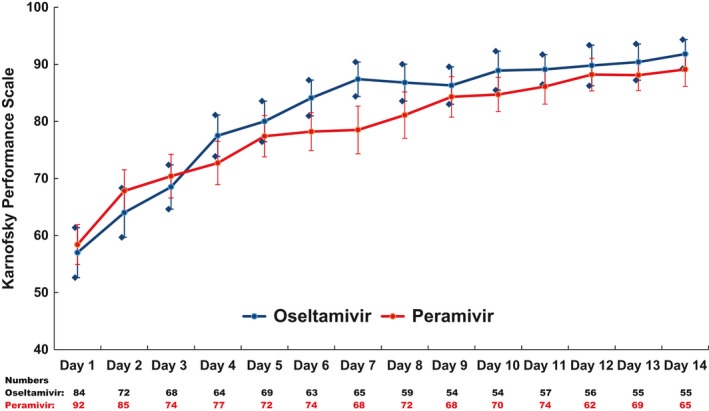
Karnofsky performance scale by day. The blue diamonds represent the lower and upper bound of a 95% confidence interval of each point estimate of the Karnofsky Performance Scale of patients in the oseltamivir group; the red bars represent the lower and upper bound of a 95% confidence interval of each point estimate of the Karnofsky Performance Scale of patients in the peramivir group

For assessment of outcome measurement, chart review was performed on all 179 participants (peramivir: 95; oseltamivir: 84) and daily follow‐ups for peramivir and oseltamivir groups were obtained from 88 and 79 participants (day 1), 77 and 70 patients (day 3), 70 and 66 patients (day 7), 68 and 57 patients (day 14), and 86 and 72 patients (day 28), respectively (Figure 1). The average FLU‐PRO score at baseline was similar between the two groups (peramivir: 2.67 vs oseltamivir: 2.52) and scores consistently decreased over time for both groups (day 5: peramivir: 1.71 vs oseltamivir: 1.62; day 10: peramivir: 1.48 vs oseltamivir: 1.37; day 14: peramivir: 1.40 vs oseltamivir: 1.33; all *P* < .05 for significantly non‐inferior) (Figure 2). PGIS score at baseline was also similar between the two groups (moderate or severe symptoms: peramivir: 82% vs oseltamivir: 84%) and the score decreased over time for both groups (day 7: peramivir: 27% vs oseltamivir: 18%; day 28: peramivir: 2% vs oseltamivir: 7%) (Figure 3). Regarding patient's clinical status, Ordinal Scale scores declined from day 1 to 14 for both groups (day 1 – peramivir: 3.0 vs oseltamivir: 3.0; day 7 – peramivir: 1.6 vs oseltamivir: 1.4; day 14 – peramivir: 1.3 vs oseltamivir: 1.3; all *P* < .05 for significantly non‐inferior) (Figure 4). At the same time, the Karnofsky performance scale measures increased for both groups (peramivir: 58.4 vs oseltamivir: 57.0) from the high 50s at day 1, to approximately 80 at day 5 (peramivir: 77.4 vs oseltamivir: 80.0) and approximately 90 at day 14 (peramivir: 89.1 vs oseltamivir: 91.8) (Figure 5). Daily Karnofsky performance scale of the peramivir group was not inferior to that of the oseltamivir group except for at day 7 (78.5 vs 87.4) and 8 (81.1 vs 86.8). Results of sensitivity analysis showed the same results that the outcomes of peramivir group were not appreciably worse than those in oseltamivir group except for Karnofsky performance scale at day 7 (data not shown). Clinical course of the peramivir group was not inferior to that of the oseltamivir group by ED disposition (admission or discharge) evaluating by daily FLU‐PRO, Ordinal Scale, PGIS score or Karnofsky performance scale except for at day 6 and 7 for hospitalized patients. Of note, there were no statistical differences in these four indicators between those with onset of symptoms greater 2 days and those less than 2 days, by treatment group, except for day 2 Ordinal Scale in oseltamivir group (onset ≤2 days: 2.7 vs >2 days: 2.5, *P* = .034).

Regarding patients with more than one CDC high‐risk factor for an influenza complication, they did not fare worse than those with only one factor by either group in the PGIS score and the Ordinal Scale score by day. However, they reported their physical activities were worse than those with only one factor in the Karnofsky performance scale by both treatment groups in most of days followed (peramivir: day 2‐11; oseltamivir: day 2‐3, day 7‐10, day 12‐13). On the other hand, they reported their symptoms were getting much better on certain days after enrollment according to their FLU‐PRO score than those with only 1 factor (peramivir: day 5, day 8‐11; oseltamivir: day 2‐3). Regarding the impact of medical history of chronic pulmonary diseases on the study outcomes, patients treated with oseltamivir who had chronic pulmonary diseases did not fare worse than those without chronic pulmonary diseases, according to their FLU‐PRO, PGIS score, Ordinal Scale score, or the Karnofsky performance scale by day. The same trend was observed in the peramivir group, with the exception of the Karnofsky performance scale between day 2 to 7, and at day 10. Regarding the impact of medical history of metabolic diseases, there were no differences in Ordinal Scale score in either treatment group. There were also no differences in terms of inferiority in other three indicators for the majority of days patients were followed in either group (data not shown).

**FIGURE 6:**
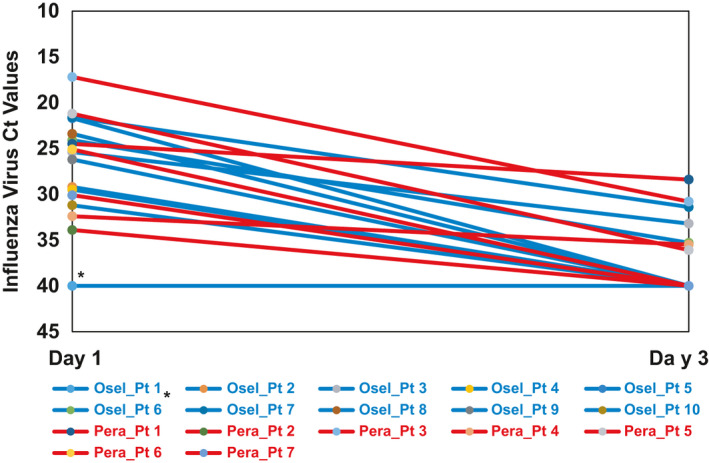
Influenza virus cycle threshold (C*_t_*) values at day 1 and 3 among 17 influenza‐infected participants by antiviral treatment group. Influenza virus cycle threshold (C*_t_*) value, which is inversely proportional to the amount of influenza virus nucleic acid target in the sample, represents the number of cycles it takes to yield a positive value in quantitative Cepheid GeneXpert® Xpress Flu/RSV real‐time PCR assay. A C*_t_* value of 40 for influenza virus testing was considered as an undetectable viral load for influenza virus. Each red line represents specific individual participant who was in peramivir treatment group and each blue line represents participant who was in oseltamivir treatment group. * Clinical testing by Xpert Flu at the enrollment (day 1) for this patient was positive with influenza A virus. A C*_t_* value of 40 of the aliquoted stored clinical specimen by the *Cepheid GeneXpert® Xpress Flu/RSV real‐time PCR assay* indicated the possible degradation of the archived sample

Among the 17 paired samples, 10 were in the oseltamivir group and seven were in the peramivir group. One patient in the oseltamivir group had an undetectable viral load at both time points, even though clinical testing by Xpert Flu at the enrollment was positive with influenza A virus. Of note, before coming to the study ED, this patient had symptoms of shortness of breath, fever and chill for 3 days and had been diagnosed with pneumonia and treated with antibiotics in another hospital the day before. This patient also tested positive for a second pathogen, respiratory syncytial virus, at the time of enrollment and with testing of aliquoted samples from day 1 and 3. Of the remaining 16 patients, influenza viral load at day 3 dropped to undetectable (ie, C*_t_* = 40) in nine patients (peramivir: 3; oseltamivir: 6) (Figure 6). Of the remaining seven patients, C*_t_* values significantly increased from day 1 to 3 in all seven patients (peramivir: 4; oseltamivir: 3). On average, the C*_t_* values for the peramivir group and oseltamivir group were 26.3 and 27.2 at day 1, respectively and were 35.8 and 38.0 at day 3, respectively.

Influenza‐related complications were similar between the two groups (peramivir: 30.5% vs oseltamivir: 21.4%). The most common complication was the requirement for oxygen supplement (peramivir: 23.2%; oseltamivir: 21.4%), followed by pneumonia (peramivir: 11.6%; oseltamivir: 14.3%) and admission to ICU (peramivir: 2.1%; oseltamivir: 0%). There was no difference in preventing clinical diagnosed secondary bacterial pneumonia by treatment group (peramivir: 5.3%; oseltamivir: 4.8%, *P* = .878). One patient in the IV peramivir group had a myocardial infarction but none needed extracorporeal membrane oxygenation or had a stroke. There were no deaths occurring in either group of patients during the 28‐day follow‐up period.

Overall, there were a total of 311 adverse events reported (peramivir: 159, oseltamivir: 152), which included 14 serious adverse events from 116 patients (peramivir: 61, oseltamivir: 55). Among them, 186 (peramivir: 90, oseltamivir: 96) or (1.04 event per patient; peramivir: 0.95; oseltamivir: 1.14) were related to the study products from 87 patients (peramivir: 43, oseltamivir: 44), but none of these were categorized as serious adverse events. The most common relevant adverse event for the peramivir group patients was diarrhea (n = 28, 31.1%), followed by insomnia (14.4%), nausea (12.2%), vomiting (11.1%) and vertigo (11.1%) while diarrhea (n = 25, 26.0%) was the leading relevant adverse event for the oseltamivir group patients, following by nausea (17.7%), vomiting (15.6%), insomnia (11.5%), and vertigo (7.3%).

## 4 DISCUSSION

In this first ED‐based randomized controlled influenza therapeutic clinical trial that fully enrolled, randomized, and initiated antiviral treatment intervention in EDs to compare outcomes of patients treated with IV peramivir vs a 5 day of oral oseltamivir, we found that the regimens were similar with regard to patient's self‐reported relief of influenza symptoms, reduction of functional impairment, as well as the rates of adverse and severe adverse events, for influenza‐infected CDC categorized “high‐risk” patients. Consistent with prior peramivir vs oseltamivir randomized trials in hospitalized or outpatients, our trial in ED patients provides similar findings with regard to the clinical efficacy and safety of the use of single‐dose IV peramivir.^13^, ^15^, ^16^ To the best of our knowledge, this study provides the first evidence‐based findings for use of IV peramivir in patients who present to an ED setting. As the majority of patients who are considered high risk for influenza complications receive intravenous lines as part of their ED care, the added burden of administration of IV peramivir would be unlikely to have a significant negative effect on ED staff work burden, or ED patient flow.

In this study, we employed several validated symptom, disease severity, clinical, and physical functionality indexes to evaluate the outcomes associated with peramivir and oseltamivir treatment. All of them pointed to the same conclusion, namely that influenza symptoms were mitigated, disease severity decreased, and clinical and physical functionality improved over time with single‐dose IV peramivir administered in the ED; further these outcomes were functionally similar to those observed among the group treated with a 5‐day course regimen of oseltamivir. This finding, supports findings from previously observational studies conducted in non‐ED studies,^13^, ^15^, ^16^ but also provides important direct data for ED clinicians, to support consideration of single‐dose treatment for influenza‐infected patients at increased risk for influenza‐related complications as an alternative to oral oseltamivir. Given the busy, episodic nature of the ED, and the fact that compliance with medications at the time of discharge in some ED populations may be challenging, this additional therapeutic option may be appealing to ED providers and patients. It is important to note, however, that the current costs of peramivir are 6‐time higher than a 5‐day course of oseltamivir.^24^ Further investigations are thus warranted, taking into account issues of adherence to oseltamivir, and assessment of other factors that could be impacted by treatment compliance, including emergence of resistant strains and spread of partially treated disease in the community.

As noted above, one of the potential advantages of the use of a single‐dose regimen of antiviral medication for influenza treatment, is that patients might be less likely to adhere to a multiple‐day multiple dosages (eg, 5‐day course of oseltamivir).^25^ Our data upheld this conjecture, since approximately 20% of patients in the oseltamivir group did not adhere to the full‐course of treatment; rates of non‐adherence would likely be even lower in the real‐world setting (where patients have to fill and pay for their own prescriptions, vs here, where subjects were provided with the actual medications at the time of enrollment). Another study in Spain also demonstrated relatively low rates of adherence to oseltamivir during both pandemic and non‐pandemic influenza seasons.^26^ Non‐adherence is particularly concerning during influenza pandemics when the virus may be more virulent and/or more likely to spread in the population. Single‐dose peramivir, the recently approved baloxavir, or recently recommended use of one‐dose or two‐doses intravenous zanamivir by European Medicines Agency's Panel,^27^ thus provide added potential value for the population, which would be particularly important during a pandemic.

Of note, more than 50% of our enrollees reported onset of the respiratory symptoms more than 48 hours before coming to the ED, consistent with our previous study, as well as others ED‐based studies.^28^, ^29^ Our results demonstrate that antiviral treatment for those with greater than 2 days of symptoms also benefit from therapy in both treatment groups since the clinical aspects of improvement by all indices measured in this study were similar regardless of duration of symptoms within or greater 48 hours. Further investigation of the impact of antiviral medication on influenza patients with longer duration of symptoms could provide additional evidence for guiding future CDC treatment recommendations regarding timing of treatment initiation (relative to symptom onset). Based on our findings here, EDs could represent an important clinical venue for conducting this type of research in the future.

One of the important features of our study is that we recruited a substantial numbers of minority influenza‐infected patients to this randomized controlled trial. Approximately, two‐thirds (67%) of participants were African American and 16% were Hispanic ethnicity. Studies have documented racial and ethnic disparity regarding influenza vaccination and influenza‐related hospitalization.^30^, ^31^, ^32^, ^33^, ^34^ On the other hand, little data is available in the literature related to antiviral treatment association with race/ethnicity. Only one study surveying the perceived acceptance of peramivir which was under emergency use authorizations during the 2009 H1N1 influenza pandemic, found that African American had the lowest willingness to accept the new antiviral for influenza treatment as compared to other racial/ethnic groups.^35^ Notably, a previous systematic literature review on antiviral chemoprophylaxis against pandemic and seasonal influenza did not address the issue of potential differences in racial/ethnic group response to antiviral treatment.^35^ Our capability to recruit a considerable number of minority patients to an influenza therapeutic randomized clinical trial provides a stepping stone for future studies and could help minimize disparities associated with antiviral treatment studies in minority populations and increase acceptance of use of antiviral among minority populations.

There are a number of limitations associated with this study. First, this study was not powered to determine the overall efficacy of peramivir in treating high‐risk ED patients with influenza, as the primary aim of the study was to determine the feasibility of conducting influenza‐related therapeutic clinical trials in the ED setting. Second, the outcomes of antiviral treatment might be influenced by the virulence of influenza virus as well as its antiviral resistance level by each influenza season. However, we did not set out, to do further subtyping and/or characterization of antiviral resistance for this study. Third, even though self‐reported treatment outcome measures that we used have been validated, information bias, rooted in self‐reported data could have differentially occurred between the antiviral treatment groups. Fourth, some information (eg, duration and amount of antipyretic use) which might be associated the outcome of antiviral treatment was not collected during the trial. We were thus not able to assess the impact of these variables since we were not able to go back to collect that information. Fifth, our evaluation of influenza viral loads before and after administration of antivirals for this study used stored aliquoted samples, which could have suffered from degradation of the archived samples, especially for those with low viral load. Finally, it is also possible to have biases arising from missing data in the patient daily diary reports, and loss to follow‐ups in this study.

In conclusion, in this ED randomized controlled clinical trial, we found the clinical and physical functionality outcomes of one‐dose IV‐administered peramivir was comparable to 5‐day course oral oseltamivir for CDC‐defined “high‐risk” influenza patients. Influenza‐related complications were minimal and side effects relevant to antiviral medication were mild and infrequent in both groups. While further cost‐effectiveness studies are required, ED clinicians should consider the option of single‐dose IV‐administered peramivir for treating influenza‐infected ED patients, especially those who already have intravenous lines in place.

## CONFLICT OF INTEREST

RER reports personal fees from Cepheid Inc, grants from Cepheid Inc, during the conduct of the study; personal fees from Roche Molecular, grants from Janssen, and grants from Cepheid, Inc, outside the submitted work. All other authors report no potential conflicts.

## AUTHOR CONTRIBUTIONS

Yu‐Hsiang Hsieh: Data curation (supporting); Formal analysis (lead); Investigation (supporting); Methodology (supporting); Validation (supporting); Visualization (lead); Writing‐original draft (lead); Writing‐review & editing (lead). Andrea Dugas: Conceptualization (equal); Data curation (lead); Funding acquisition (lead); Investigation (lead); Methodology (lead); Project administration (lead); Writing‐review & editing (supporting). Frank LoVecchio: Data curation (supporting); Investigation (supporting); Project administration (supporting); Supervision (lead); Writing‐review & editing (supporting). Breana McBryde: Data curation (supporting); Investigation (supporting); Project administration (supporting); Writing‐review & editing (supporting). Erin Ricketts: Project administration (lead); Resources (supporting); Supervision (supporting); Writing‐review & editing (supporting). Kathryn Shaw‐Saliba: Investigation (supporting); Methodology (supporting); Supervision (supporting); Validation (supporting); Writing‐review & editing (supporting). Richard E Rothman: Conceptualization (equal); Funding acquisition (lead); Investigation (lead); Methodology (lead); Project administration (lead); Resources (lead); Supervision (lead); Validation (lead); Visualization (supporting); Writing‐review & editing (lead).

## ETHICAL APPROVAL

Maricopa Medical Center, Phoenix, Arizona and The Johns Hopkins University School of Medicine Institutional Review Board approved the study.

*Trial Registration*: NCT02609399 “ED Influenza Therapeutic Pilot Study: Oseltamivir vs Peramivir”.
